# Lactobacillus Genus Complex Probiotic-Induced Changes on the Equine Clitoral Microbiome

**DOI:** 10.3390/vetsci12030232

**Published:** 2025-03-03

**Authors:** Fiona Herzog, Kassandra R. Crissman, Kalie F. Beckers, Guoli Zhou, Chin-Chi Liu, Jenny L. Sones

**Affiliations:** 1 Veterinary Clinical Sciences, School of Veterinary Medicine, Louisiana State University, Baton Rouge, LA 70803, USA; fionaherzog@hotmail.com (F.H.); kcriss1@lsu.edu (K.R.C.); kbeckers@tulane.edu (K.F.B.); 2 Center for Statistical Training & Consulting (CSTAT), Michigan State University, East Lansing, MI 48824, USA; zhoug@msu.edu; 3 Office of Research and Graduate Education, School of Veterinary Medicine, Louisiana State University, Baton Rouge, LA 70803, USA; cliu@lsu.edu; 4 Clinical Sciences, Colorado State University College of Veterinary Medicine and Biomedical Sciences, Fort Collins, CO 80423, USA

**Keywords:** equine, microbiome, reproduction, clitoris

## Abstract

Dysbiosis of the lower reproductive tract (LRT) in mares may play a role in clinical diseases, including endometritis and placentitis. Metagenomic/metagenetic analysis of bacterial DNA can identify organisms that are not readily cultured and, thus, may go undetected. In this study, we tested the following hypotheses: (1) the clitoris of estrual mares harbors a unique resident microbiome, (2) topical Lactobacillus genus complex (LGC)-containing probiotic will alter the equine clitoral microbiome, and (3) early pregnancy rates following clitoral LGC application will not differ significantly from industry standards. Mares (n = 12) in estrus had sterile clitoral swabs collected (0) prior to daily topical LGC for 4 days. Second (12 h) and third clitoral swabs (48 h) were collected following final LGC application. During the next estrus, the mares were bred by artificial insemination. Genomic DNA was extracted and used for 16S rRNA sequencing via the Illumina Miseq platform. Abundance was evaluated via Friedman test with pairwise Dunn’s post hoc comparisons. Statistical significance was set at *p* < 0.05. Compared to time 0, Desulfobacterota decreased and *Corynebacterium* spp. increased at 12 h and 48 h compared to 0, while *Actinobacillus* and *Fusobacterium* spp. increased in a time-dependent manner. Furthermore, *Mobiluncus* spp. and Christensenellacea_R-7_group decreased at 12 h and 48 h compared to 0. LGC changed the beta but not alpha diversity at both 12 h and 48 h. Mares with LGC application achieved an 85% pregnancy rate in the subsequent estrus. Future investigations are needed to understand the role of the LRT microbiome and probiotics in equine breeding.

## Simple Summary

The equine lower reproductive tract (LRT) harbors a community of commensal organisms that, when altered, may contribute to pathology in mares. Recent metagenetic studies of bacterial DNA content have shown the equine LRT, as well as the uterus and placentae of the mare, accommodate micro-organisms in varying diversity, abundance and richness. The present study tested the result of local application of Lactobacillus genus complex (LGC) probiotic gel on the equine clitoral microbiome in estrual mares. The top three most abundant phylum residents in the equine clitoral fossa before LGC were Firmicutes, Bacteroidota and Fusobacteriota, contradictory to the equine uterine and vaginal microbiomes, with Proteobacteria in the top three phyla present. At the genus level, *Lactobacillus* spp. increased in the clitoral fossa at 12 h but returned to pre-treatment levels by 48 h. This transient impact on the clitoral microbiome in mares was associated with more sustained changes in the abundance of other bacterial species observed at 48 h. The LGC-treated mares had an 85% pregnancy rate in the following estrus. Our results indicate that the equine clitoral fossa is distinct from the vaginal and uterine microbiomes, as measured by metagenetics. LGC application dynamically alters the resident clitoral microbiome but does not interfere with the fertility of healthy mares.

## 1. Introduction

The equine lower reproductive tract harbors a community of commensal organisms that have been evaluated using traditional cultivation techniques [1,2]. Dysbiosis within this community may play a role in clinical diseases of the mare, including endometritis, metritis and placentitis.

Metagenetic analysis of bacterial DNA can identify bacteria that are not readily cultured in standard aerobic conditions [3], and estimates of bacterial abundance at reproductive sites utilizing cultivation techniques must be extrapolated from in vitro growth under laboratory conditions. Therefore, metagenetic analysis may provide a more sensitive and in situ evaluation for bacterial community presence. Metagenetic studies have discovered microbiomes within the placenta [4,5,6], vagina [7,8,9,10] and uterus [8,9,10,11,12,13,14,15,16,17,18,19] of the mare. Recently, the clitoral fossa microbiome has been described in maiden mares [8]. While it shared predominant phyla with the uterus and vagina, the equine clitoral fossa of maiden mares clustered independently, and the beta diversity was significantly different from other reproductive sites. Our study is the first to evaluate the changes induced by probiotic treatment on the equine clitoral microbiome.

The clitoris has been described as a potential reservoir of bacteria that may contaminate the upper reproductive tract during breeding or routine reproductive procedures [1,20,21]. Previous studies suggest that the equine uterine microbiome is influenced by lower reproductive tract sites during reproductive states associated with a relaxed, patent cervix [9,10,12]. Similarly, in human females, graduated shifts in bacterial community composition from the vagina, cervix, endometrium and uterine tubes are observed [22]. In this regard, it is possible that dysbiosis of the lower reproductive tract may predispose the uterus to colonization with pathogenic bacteria. Additionally, bacterial species *Taylorella equigenitalis*, *Pseudomonas aeruginosa* and *Klebsiella pneumoniae*, which may harbor within the equine clitoris, present a risk of venereal transmission [21,23,24,25]. Understanding the normal clitoral microbiome may present opportunities for infectious reproductive disease management and prevention.

LGC-containing probiotics have been evaluated for their benefit in the treatment of vaginal conditions in women [26,27,28,29,30,31,32,33], and it is possible they may confer similar probiotic changes to promote reproductive health in mares. Lactic acid bacteria (LAB), including LGC bacteria, harvested from the reproductive tract of mares, have demonstrated in vitro probiotic potential, including biofilm formation, adhesion to vaginal epithelial cells, co-aggregation, auto-aggregation and the ability to inhibit the growth of known reproductive tract pathogens, including *Streptococcus equi* subsp. *Zooepidemicus* and *Escherichia coli* [34,35]. The benefits of topical vaginal application of LGC probiotic formulas have also been evaluated in dairy cows, with improvements in clinical parameters related to post-partum metritis and endometritis [36,37,38]. Therefore, this study will aim to present a descriptive analysis of the mare clitoral microbiome, in addition to appraising the reproductive safety and changes induced by a commercially available LGC probiotic on the clitoral microbiome. The LGC-containing gel used in this study is similar in composition to a formula previously evaluated for its effects on the vaginal microbiome and in vitro and in vivo efficacy against vulvovaginal candidiasis (VVC) in women [32]. It is hypothesized that the clitoris of the mare will harbor a unique microbiome, which will be altered following application of LGC-containing probiotic. The observed alteration may be reflected by increased amounts of *Lactobacillus* spp. but also the change in relative abundance of lactic-acid-sensitive bacteria in the clitoral fossa of mares. Additionally, per-cycle early pregnancy rates will be assessed when mares are bred on the following estrus cycle after treatment to determine an absence of negative impacts to conception and early pregnancy maintenance.

## 2. Materials and Methods

### 2.1. Mare Selection

This study’s protocol was approved by the Louisiana State University Institutional Animal Care and Use Committee: approval number, 21-159; approval date, 8 February 2022. All the horses included in this study were housed on adjacent pastures. The mares received a pelleted ration, in addition to free choice pasture. A total of 14 reproductively normal mares, of Thoroughbred and Quarter Horse breeds, aged 4–22 years (median age, 7 years), were selected based on the following criteria:

(a) Absence of abnormalities on reproductive palpation, ultrasound and vaginal speculum examination performed during estrus. (b) Absence of bacterial growth (endometrial swab) or reproductive pathogens (clitoral swab) on aerobic cultures performed on endometrial samples collected by double-guarded endometrial swab (Minitube, Verona, WI, USA) and swabs collected from the clitoral fossa during estrus. (c) Absence of inflammation on endometrial cytology collected by double-guarded endometrial cytobrush (Minitube, Verona, WI, USA), indicated by <2% neutrophil across 10 high-power field [39]. (d) Absence of clinical signs of endometritis (uterine fluid during diestrus, >2 cm uterine fluid during estrus, inappropriate or excessive endometrial edema) during examinations performed every one to three days, for one complete estrous cycle.

### 2.2. Sample Collection

Samples were collected from the clitoral fossa by everting the clitoris and manipulating a sterile culturette swab through the medial and lateral aspects of the clitoral recess and in the region of the clitoral sinus. The swab tip was pre-moistened with sterile saline solution to aid in bacterial DNA uptake. If gross fecal and mud contamination was present on the perineum, this was removed with a dry paper towel prior to sample collection. The samples were stored at −80 °C. At each sampling timepoint, control samples were also collected to evaluate environmental contamination by waving a sterile, pre-moistened culturette swab in the vicinity where the mare was positioned for sample collection. The control samples were stored and processed for DNA extraction and quality control. A total of five control samples were randomly selected for microbiome sequencing.

### 2.3. LGC Probiotic Gel

The LGC-containing probiotic (VGN Probiotic Intimate Gel, YUN NV) was a silicone-based gel containing two strains of LGC: *Lactiplantibacillus pentosus* YUN-V1.0 and *Lactiplantibacillus plantarum* YUN-V2.0 (concentration of LGC bacteria 10^9^ CFU/gram, gel density: 0.94 +/− 0.05 g/mL). The specific ingredients of the gel are listed as follows: dimethicone, dimethicone/vinyl dimethicone crosspolymer, lactobacilli.

### 2.4. Microbiome Sampling and Probiotic Treatment

The mares were examined by rectal palpation and ultrasound as needed to monitor follicular development and daily to every second day during estrus. Pre-treatment samples (0 h) were collected when a mare was first determined to be in estrus (presence of a ≥ 25 mm follicle, ≥2 endometrial edema, relaxed cervix). Topical probiotic treatment was then initiated by applying 1 mL probiotic gel to the everted clitoral fossa once daily for four days with a sterile 3 mL syringe. Post-treatment samples were then collected at approximately 12 h (12 h) and 48 h (48 h) following the final probiotic treatment. The mares were then administered dinoprost 10 mg (Lutalyse, Zoetis) intramuscularly at day 7–10 post-ovulation following treatment and sampling to induce the next estrus for the breeding trial.

### 2.5. Post-Treatment Breeding Trial

When a pre-ovulatory follicle (≥35 mm diameter) with appropriate endometrial edema (≥2) was observed, the mares were bred with 1 billion progressively motile spermatozoa by artificial insemination into the uterine body. Ovulation induction was performed with 2000 IU hCG (Chorulon, MSD Animal Health) administered intravenously 0–24 h prior to breeding. Ovulation was confirmed 24–48 h post-breeding, and early pregnancy diagnosis was performed 13–15 days post-ovulation by rectal palpation and ultrasound. A second swab was collected from the clitoral fossa for aerobic culture prior to breeding to confirm the absence of reproductive pathogens following treatment.

### 2.6. DNA Extraction and Quality Control

DNA Extraction: Bacterial DNA extraction was performed using Qiagen QIAamp PowerFecal Pro DNA Kit extraction kits (Qiagen, Germantown, MD, USA) according to the manufacturer’s protocol. Two additional control samples were prepared by performing DNA extraction using only the kit contents to assess for contamination derived from DNA extraction procedures. The presence of the 16srRNA gene and DNA quality were evaluated using PCR and gel electrophoresis prior to metagenetic sequencing. The V4 variable region of the 16s rRNA gene was amplified with PCR primers 515f/806r in a 30-cycle PCR using the DreamTaq Hot Start PCR Master Mix Kit (Thermoscientific, Waltham, MA, USA). The PCR products were evaluated on a 2% agarose gel for correct product size formation (approximately 350 bp) against a 100 m bp DNA Ladder (Thermoscientific, Waltham, MA, USA). The results of the PCR DNA quantification and gel electrophoresis were consistent with no to minimal environmental contamination.

### 2.7. DNA Sequencing and Data Analysis

A total of 42 clitoral samples and 7 control samples were submitted to Michigan State University for microbiome sequencing. Michigan State University Genomics Core prepared amplicon libraries of the bacterial 16S-V4 hypervariable region using dual-indexed Illumina-compatible fusion primers 515f/806r, as described by Kozich et al. [40]. The sequencing data were analyzed using Qiimi2 Phyloseq Bioinformatics Pipeline for 16S Microbiome Data. For amplicon processing, rarefaction was performed, and the samples were excluded based on a minimum of 1000 reads. At the conclusion of rarefaction, all the blank samples and two mare subjects were removed from the analysis. The raw sequence data from clitoral samples of 12 mares were imported to QIIME2 (qiime2-amplicon-2023.9) for processing. The samples were denoised and trimmed using DADA2 pipeline, and taxonomy was assigned using a pretrained classifier (silva-138-99-515-806-nb-classifier.qza). Members of LGC were sequenced and reported at the genus level as *Lactobacillus* by the pretrained classifier. It should be noted that recent re-classification has divided the former genus of *Lactobacillus* into 25 genera. However, ‘lactobacilli’ may remain a useful term to describe all organisms that were classified as the family *Lactobacillaceae* up until 2020 [41]. The results of this study and previous studies relating to LGC bacteria sequenced as ‘*Lactobacillus*’ will be referred to as such.

The R package ‘phyloseq’ was applied for diversity analysis, and rarefaction was used to normalize the library sizes across the sequences. Alpha diversity metrices observed and Shannon were calculated, followed by bar-plotting and pairwise Wilcoxon rank-sum test. Beta diversity was analyzed using the Bray–Curtis distance metric, followed by PCoA plotting and permutation multivariate analysis of variance (PERMANOVA). For analysis of differentially abundant taxa to the species level between timepoints, the DESeq2 R package was applied with un-rarefied data. Sparse features (>90% zeros across the samples) were pruned, and the data were normalized using the EstimateSizeFactors function in the DESeq2 package with the ‘poscounts’ method. Significance for differential abundance and diversity analysis was set at a *p* adjusted value of 0.05 using Benjamini and Hochberg false discovery rate correction. Graph Pad Prism (Windows, Version 10.1.2, GraphPad 10.2.20 (392) Software, LLC) was used for relative abundance analysis and to generate figures. Significant differences in top 10 phyla and top 25 genera were evaluated via the Friedman test with pairwise Dunn’s post hoc comparisons, whereby the significance was determined at 0.05.

## 3. Results

### 3.1. Taxonomic Abundance

**Figure 1:**
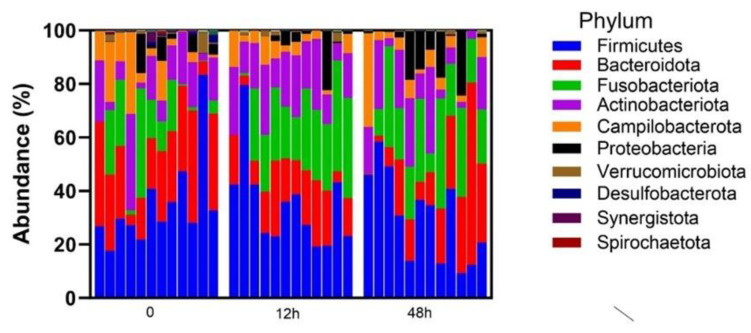
Relative abundance of top 10 phyla in the clitoral microbiome by sample. Time 0 = pre-treatment with LGC-containing probiotics, time 12 h = 12 h post-LGC treatment, time 48 h = 48 h post-LGC treatment.

**Figure 2:**
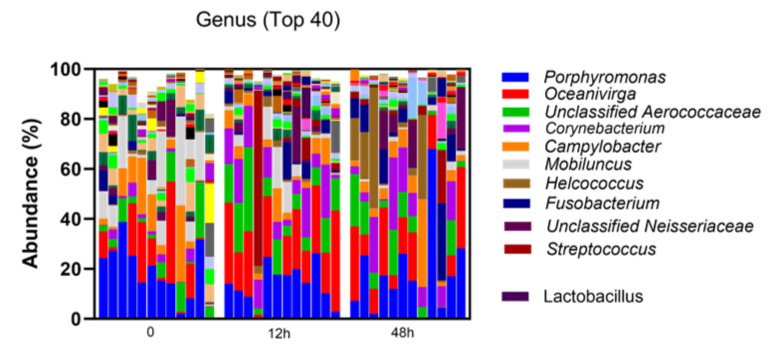
Relative abundance of top 25 genera in the clitoral microbiome by sample. Time 0 h = pre-treatment with LGC-containing probiotics, 12 h = 12 h post-LGC treatment, 48 h = 48 h post-LGC treatment. The color key identifies the proportion of the top 10 most abundant genera, *Lactobacillus* and remaining top 14 genera as ‘Others’.

A total of 36 clitoral samples were analyzed; two mares (6 samples) were excluded due to insufficient reads from the 42 total samples collected, generating 872 total operational taxonomic units. The clitoral microbiome was dominated by Firmicutes, Bacteroidata and Fusobacteriota at the phylum level (Figure 1). The top 10 genera in descending order were Porphyromonas, Oceanivirga, unclassified Aerococcaceae, Corynebacterium, Campylobacter, Mobiluncus, Helcococcus, Fusobacterium, unclassified Neisseriaceae and Streptococcus (Figure 2).

**Figure 3:**
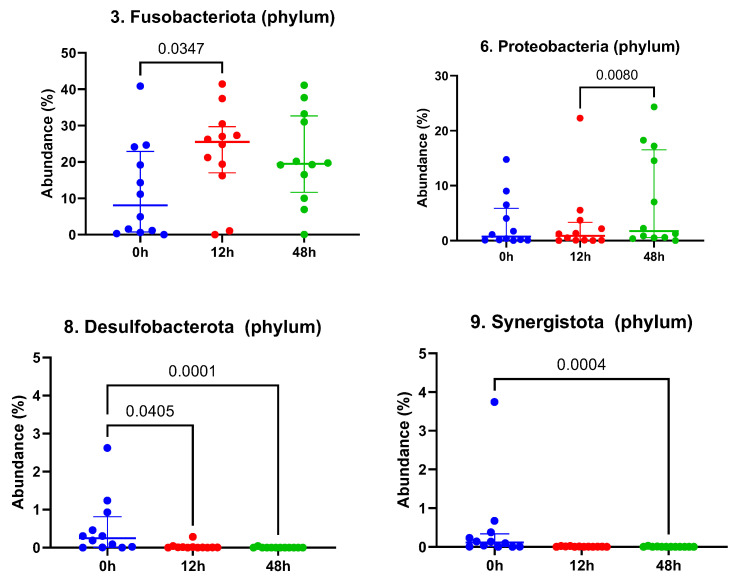
Quantification of relative abundance for Fusobacteriota, Proteobacteria, Desulfobacterota and Synergistota phyla before and after treatment with LGC-containing probiotics. Time 0 = pre-treatment, 12 h = 12 h post-LGC treatment, 48 h = 48 h post-LGC treatment. Solid line with *p* value identifies significant differences. Numbers in figure titles are indicative of phylum overall abundance.

When evaluating the top 10 phyla, significant differences were observed following the LGC-containing probiotic treatment. The taxonomic abundance of Fusobacteriota was seen to increase significantly between time 0 and 12 h post-treatment (*p* = 0.035, Figure 3), while the Proteobacteria increased significantly from 12 h to 48 h post-treatment (*p* = 0.008, Figure 3). Desulfobacterota and Sinergistota were observed to decrease from pre-treatment values (Figure 3) at time 12 and 48 h post-treatment (*Desulfobacterota*, *p* = 0.041 and *p* < 0.001, respectively) and 48 h post-treatment (Sinergistota, *p* < 0.001).

**Figure 4:**
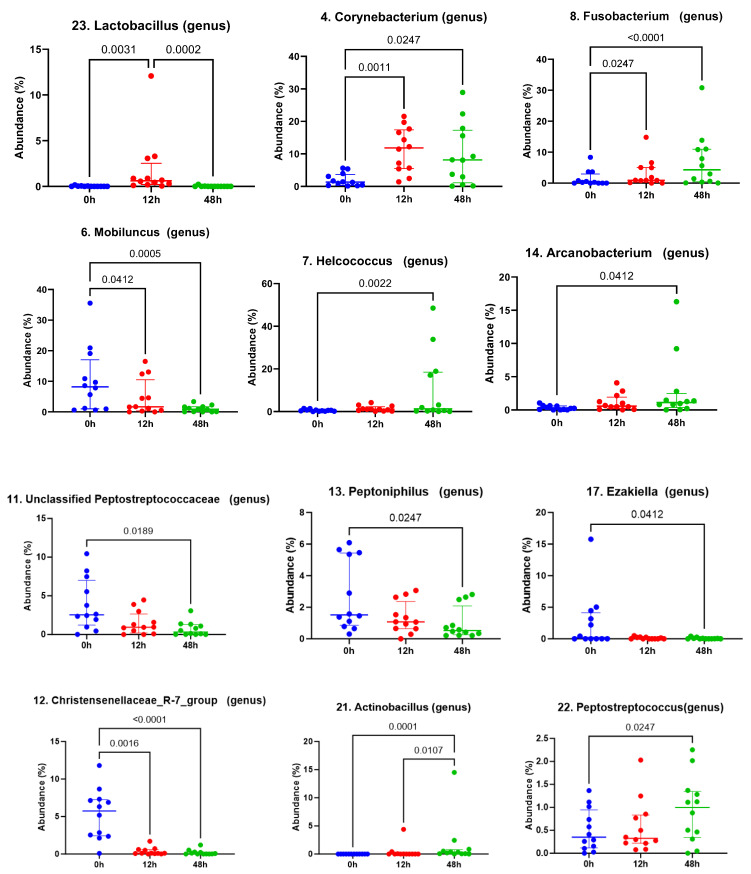
Quantification of relative abundance for significantly different genera before and after treatment with LGC-containing probiotics *Lactobacillus*, *Corynebacterium*, *Mobiluncus*, *Fusobacterium*, *Helcococcus*, *Arcanobacterium*, unclassified *Peptostreptococcaceae*, Christensenellaceae R-7 group, *Peptinophilus*, *Ezakiella*, *Actinobacillus*, *Peptostreptococcus*. Time 0 = pre-treatment, 12 h = 12 h post-LGC treatment, 48h= 48 h post-LGC treatment. Solid line with *p* value identifies significant differences.

Evaluation of the top 25 genera additionally presented significant differences, which are depicted in Figure 4. In particular, Lactobacillus (the twenty-third most abundant genus) significantly increased at 12 h post-treatment with LGC-containing probiotics compared to time 0 and 48 (*p* = 0.003 and 0.0002, respectively). The mean relative abundance of Lactobacillus was found to be 0.026, 1.874 and 0.021% at time 0 (pre-treatment), 12 h and 48 h, respectively (Table 1). One mare was noted to have an increase in the relative abundance of Streptococcus genera at time 12 h post-treatment (Figure 2); however, no significant differences were observed in the Streptococcus relative abundance between timepoints. In the interest of investigating the likelihood that this may represent the reproductive pathogen, *S. equi* subsp. *zooepidemicus*, differential abundance of Streptococcus equi was evaluated at the species level across the mares and confirmed to not be significantly different following treatment (*p* adjusted: 0.32 for time 0 versus 12 h; *p* adjusted: 0.56 for time 0 versus 48 h post-treatment).

### 3.2. Measures of Alpha Diversity

**Figure 5:**
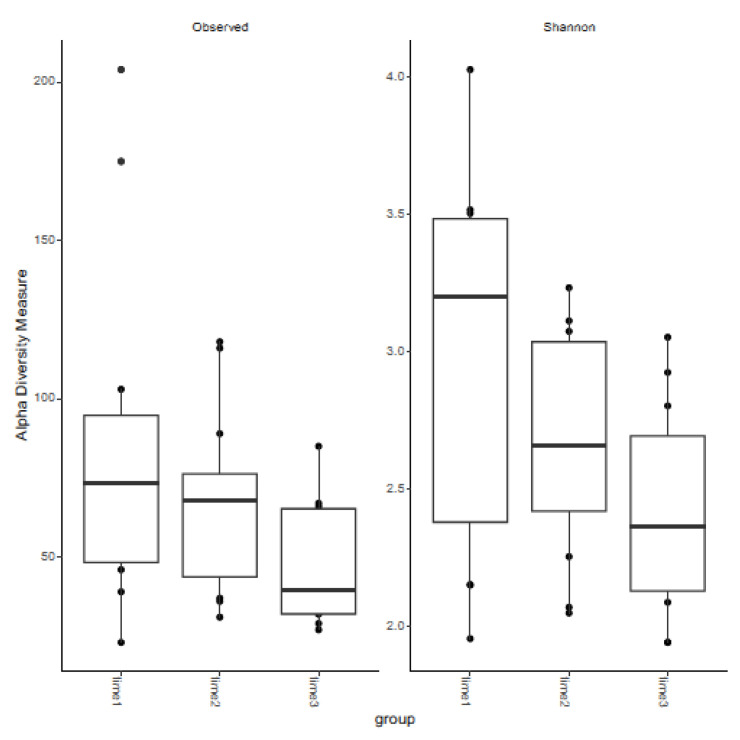
Box and whisker plots of observed and Shannon diversity indices within the equine clitoris before and after LGC-containing probiotic treatment. Time 1 = pre-treatment, Time 2 = 12 h post-treatment, Time 3 = 48 h post-treatment.

The observed and Shannon diversity indices across the three timepoints were analyzed (Figure 5). No significant differences were seen for the observed or Shannon diversity indices at any timepoint. However, differences trended toward significance between 12 and 48 h post-treatment for observed diversity (*p* adjusted: 0.096) and approached significance between time 0 and 48 h post-treatment for both observed and Shannon diversity (*p* adjusted: 0.067 and 0.072, respectively).

### 3.3. Measures of Beta Diversity

**Figure 6:**
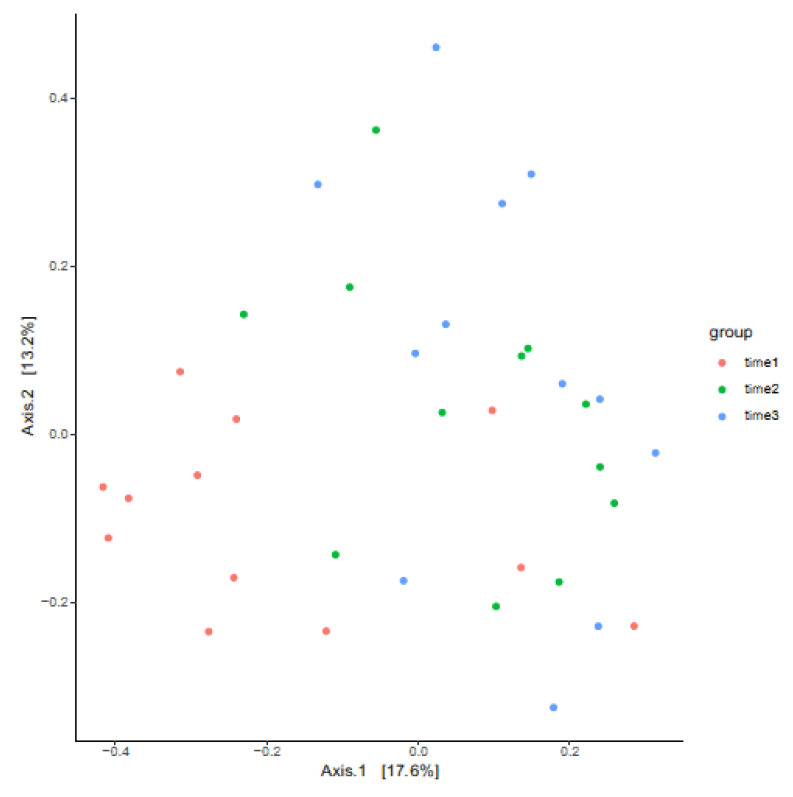
PCoA plot of beta diversity relationships between equine clitoral samples before and after LGC-containing probiotic treatment. time 1 = pre-treatment, time 2 = 12 h post-treatment, time 3 = 48 h post-treatment.

Taxonomic diversity between the groups, as determined by PERMANOVA, was unchanged between timepoints 12 and 48 h post-treatment. However, significant differences in beta diversity were apparent between time 0 and 12 h post-treatment (*p* adjusted: 0.005) and time 0 to time 48 h post-treatment (*p* adjusted: 0.003). A principal coordinate analysis (PCoA) plot shows the beta diversity relationships between the samples by sampling timepoints (Figure 6).

### 3.4. Breeding Trial Following LGC Probiotic Application

The application of LGC-containing gel was well tolerated by the mares with no negative side effects observed. Aerobic cultures of the clitoral swabs performed on the following estrus cycle after treatment confirmed the absence of reproductive pathogens. One mare was excluded from the breeding trial after ovulating unexpectedly, prior to breeding. An overall per-cycle pregnancy rate of 11/13 mares, or 85%, was achieved.

## 4. Discussion

This study describes the clitoral microbiome of the reproductively normal mare. At the phylum level, the equine clitoral microbiome was dominated by Firmicutes, Bacteroidota and Fusobacteriota. Similarly, Gil-Mirando et al. [8] found Firmicutes as the top phyla in the clitoral fossa of maiden mares, along with Bacteroidota, Proteobacteria, Actinobacteria and Epsilonbacteraeota. This is complementary to the equine vaginal microbiome, with these representing the dominant phyla in previous reports, Miranda [7,8]. At the genera level, similarities between the equine clitoral and vaginal microbiome are also apparent. For the equine clitoral microbiome, the dominant genera were *Porphyromonas*, *Oceanivirga*, unclassified *Aerococcaceae*, *Corynebacterium*, *Campylobacter*, *Mobiluncus*, *Helcococcus*, unclassified *Neisseriaceae* and *Streptococcus* (descending order of abundance). *Porphyromonas* were similarly observed to be the most abundant genera in the vagina by Barba et al. [7], while *Campylobacter*, *Corynebacterium*, *Helcococcus*, *Streptococcus*, *Mobiluncus* and *Oceanivirga* were also reported in the 15 most abundant genera.

For the endometrial microbiome, authors have frequently reported dominant phyla as Firmicutes, Bacteroidota, Proteobacteria and Actinobacteria [8,11,12,13,14], while the dominant genera appear to vary by study [12,13,14]. This may be due to differences in the geographic location of mares sampled across studies, as reported by others [13]. Based on limited references, taxonomic abundance of the clitoral microbiome appears to more closely resemble the microbiome of the vagina compared with the endometrium. This finding may support the hypothesis for graduated shifts in microbial communities from lower to upper reproductive sites in the mare, as is observed in women [22]. To determine the significance of this finding in equine pregnancy, further studies should compare the relative abundance of the dominant taxa from reproductive locations within the same mare before and after breeding.

LGC bacteria are found in significant abundance in the reproductive microbiomes of women, where evidence suggests they support reproductive health and pregnancy outcomes [3,22,42,43,44,45,46,47,48,49,50,51,52,53,54]. However, they have rarely been reported as dominant or core microbiota in mares [7,8,12,13,14]. Barba et al. [7] similarly did not detect the genus (formerly known as) *Lactobacillus* in the vagina of all mares sampled, although *Lactobacillus* was detected in at least one of two timepoints assessed (estrus and diestrus). In the present study, *Lactobacillus* (now LGC) was found in relative abundance of 0.026% prior to treatment. This is comparable to previous reports of vaginal (0.01–0.37%) [7,9] and endometrial (0.02%) microbiomes [9]. However, Holyoak et al. [13] cited *Lactobacillus* abundance as 7.5%, forming part of the core genera in the equine endometrium. In terms of prevalence, the present study detected *Lactobacillus* in 7/12 clitoral samples prior to treatment. The aim of the current study was not to replace *Lactobacillus* spp. within the clitoral fossa of mares, but rather to determine its impact on the local bacterial community structure over time.

The clitoris is known to harbor reproductive pathogens, including *Klebsiella pneumoniae*, *Pseudomonas aeruginosa* and *Streptococcus equi* subsp. *zooepidemicus*, even in normal mares [1,2]. *Streptococcus equi subsp. zooepidemicus* is a common cause of endometritis and placentitis in the mare [55,56,57,58]. *Streptococcus* has also been observed to comprise the core vaginal microbiome in mares [7]. In the mare clitoral fossa, *Streptococcus* was found to be the tenth most abundant genus. At the species level, *S. equi* was detected in the clitoris of mares prior to treatment and was not significantly altered following application of the LGC-containing probiotic gel. It was interesting to note that genera *Klebsiella* and *Pseudomonas* were not detected in any equine clitoral samples during the study period. This is in contrast to previous reports for the endometrial microbiota [12,13,14,18]. *Taylorella* was similarly not detected in any mares at any timepoint in our study. Therefore, the effect of LGC- containing probiotic gel on these known equine reproductive pathogens remains to be determined.

The present study aimed to evaluate differences in the equine clitoral microbiome following topical application of an LGC-containing probiotic gel. Studies describe the utility of LGC probiotics in the treatment of vaginal infections in women [26,27,28,29,30,31,32,33]. There is an inverse relationship between vaginal lactobacilli abundance and lower reproductive tract infections [59] and associations between endometrial or vaginal abundance and pregnancy outcomes [42,46,47,49,50,54]. The probiotic effects of LAB, including *Lactobacilli*, harvested from vaginal or vestibular samples of mares, have also been evaluated for in vitro probiotic efficacy [34,35]. For these reasons, specific changes induced by an LGC probiotic preparation on the equine clitoral microbiome were evaluated.

This study aimed to evaluate the influence of an LGC probiotic gel on the equine lower reproductive tract microbiome, with the view that probiotic treatment may have potential therapeutic applications in the future. For this reason, the persistence of LGC bacteria in the clitoral microbiome was of particular interest. The authors chose to assess 12 and 48 h after the last treatment to assess observational changes during the same estrous cycle when application may be used for improved breeding management. A significant increase in the microbial abundance of *Lactobacillus* was observed 12 h post-treatment, before returning to pre-treatment levels at 48 h post-application of the gel. It is considered that this increase most likely represents the presence of the probiotic-derived LGC. However, we were unable to sequence at the species level for *Lactobacillus* (now LGC) in this study. These results suggest that probiotic LGC bacteria persisted in the equine clitoral microbiome for 12 to 48 h and failed to colonize thereafter.

Despite the return of *Lactobacillus* abundance to baseline levels at 48 h after treatment, dynamic alterations in the equine clitoral microbiome were observed throughout the study period, specifically, anaerobic bacterial genera. Of interest, *Mobiluncus* genera were observed to decrease by 12 and 48 h post-LGC-containing probiotic treatment. *Mobiluncus* species are one of several anaerobic bacteria that are involved in the pathogenesis of bacterial vaginosis (BV) in women, where a sharp decline in LGC abundance occurs in concert with an overgrowth of BV-associated bacteria [60]. The probiotic effects of lactobacilli on another BV-associated bacteria, *Gardnerella vaginalis*, are well studied [61,62,63,64,65,66], and topical lactobacilli probiotics have been evaluated for their clinical efficacy as a treatment for BV [26,27,28,31,33]. Lactobacilli probiotics have been shown to decrease abundances of *Gardnerella vaginalis* with oral administration [67] and *Atopobium* with vaginal application [68]. In addition to this, strains of *Lactobacillus crispatus* and *Lactobacillus gasseri* have been reported to inhibit *Mobiluncus* adhesion to vaginal epithelial cells via competition, exclusion and displacement in vitro [69]. While it is possible that LGC strains in the present preparation may have exerted similar probiotic effects, further investigations are required to establish such mechanisms in the mare reproductive tract that may attenuate the colonization of pathogens. 

*Fusobacterium* genera, representing another bacterial anaerobe, was observed to increase in response to treatment at 12 and 48 h timepoints after the LGC-containing probiotic application ceased. Relationships have been demonstrated between the presence of orally associated *Fusobacterium* spp. in fetal fluids and fetal membranes, periodontal disease and adverse pregnancy events in women [70,71,72]. Orally derived bacteria have been hypothesized to affect outcomes such as premature delivery through hematogenous routes. This has been demonstrated in murine models [73,74], where intravenous injection of oral bacteria or plaque has resulted in the detection of these species within the placenta, resulting in pre-term delivery, stillbirth and non-sustained live birth. In mares, Pugh [10] evaluated alterations in oral, fecal, vaginal and uterine microbiomes in post-parturient mares. As previously mentioned, the author observed that *Fusobacterium* was two-fold higher in mares that delivered pre-term compared to full-term parturition across all the samples, suggesting that systemic increases in *Fusobacterium* were associated with early-onset parturition. Whether the increases in *Fusobacterium* seen in the present study reflect increases in orally derived bacteria is unknown. For the present population of mares, conception and early pregnancy was assessed, with pregnancy determination performed at 13–15 days of gestation. Therefore, possible implications for mid- to late-term pregnancy outcomes following topical probiotic treatment may warrant further assessment before recommending its use in pregnant mares.

Significant changes in beta diversity measures were observed between the groups in the present study following LGC-containing probiotic gel application. However, it should be noted that due to experimental design, the effect of time cannot be separated from the effect of treatment. An important consideration for time effects in the present study is the potential relationship of reproductive cyclicity and the equine clitoral microbiome composition. Previous authors have failed to detect significant differences in the vaginal microbiome when comparing estrus and diestrus mares, concluding that the vaginal microbiome is stable throughout the estrous cycle [7]. This is in contrast to the endometrial microbiome, where Heil et al. [11] observed differences in both alpha and beta diversity measures between anestrus and estrus mares. However, the time effect in that study was ~6 months between sampling mares, and the present study was only over 5 days. While it could be considered less likely that reproductive cyclicity in mares will have similar effects on the lower reproductive sites, as it does for endometrial locations, this possibility should be considered a limitation of the present study.

The length of estrus in the mare is reported as 4.5–9 days [75]. Therefore, it is not unexpected that 10 of 12 mares ovulated 24 to 120 h after beginning treatment. Administration of topical therapies in equine reproduction are commonly applied for periods of 3–7 days [76]. A treatment period of 4 days was selected to evaluate safety and potential microbiome alterations beginning in estrus when routinely given. The study mares were not kept under artificial lighting, and due to the limitations of availability in a research herd, a sham-treated cycle was not possible in any mare. The possible effect of dynamic changes in reproductive hormones surrounding the periovulatory period on the clitoral microbiome should also be considered in a future study. However, the time required for shifts in these reproductive hormones to affect alterations in microbial composition is undetermined. Further investigations are required to investigate the possible effect of reproductive hormones on the bacterial communities of the lower reproductive tract.

During the course of the treatment period, no side effects, such as pain, swelling or pruritis, were noted in any mares following application of the probiotic gel, and acceptable pregnancy rates were achieved on the first estrus following probiotic application. In the absence of apparent negative impacts to reproductive function associated with breeding, conception and early pregnancy, larger scale investigations may be indicated to further appraise the reproductive safety of this product for therapeutic use. Furthermore, intrauterine probiotic therapy will be an important future study in reproductive mares.

## 5. Conclusions

This study describes the clitoral microbiome of the reproductively normal mare before and after application of probiotics. The clitoral microbiome was dominated at the phylum level by *Firmicutes*, *Bacteroidata* and *Fusobacteriota*. Based on measures of taxonomic abundance, the clitoral microbiome more closely resembled previous reports of vaginal microbiota compared with endometrial microbiota. The gel was well tolerated by the mares, with no negative impacts to conception and early pregnancy observed when the mares were bred on the following estrous cycle post-treatment. Persistence of the LGC probiotic was evident at 12 h post-application, which returned to baseline values at 48 h following treatment. However, dynamic alterations in the relative abundance of the top phyla and genera were observed throughout the study period. Further investigations are required to determine the potential significance of these changes and confirm the reproductive safety of LGC probiotics for pregnancy maintenance and parturition.

## Figures and Tables

**Table 1:** *Lactobacillus* relative abundance (%) in equine clitoris when evaluated before and after LGC-containing probiotic treatment. Time 0 = pre-treatment, 12 h = 12 h and 48 h = 48 h post-LGC treatment.

Mare	Time 0	Time 12 h	Time 48 h
1	0.008	0.436	0
2	0.150	0.052	0
3	0.061	0.291	0
4	0	0.100	0.001
5	0	0.555	0
6	0	12.085	0.011
7	0.042	0.877	0.025
8	0	3.293	0
9	0	0.868	0
10	0.004	3.074	0
11	0.010	0.185	0
12	0.034	0.678	0.219
Mean	0.026	1.874	0.022

## Data Availability

The raw data supporting the conclusions of this article will be made available by the authors on request.
